# Nutritional Intelligence? Hyperpalatable Foods Do Not Dominate Evaluations of Familiar Snacks in a Finnish Sample

**DOI:** 10.1016/j.cdnut.2026.109553

**Published:** 2026-10-09

**Authors:** Arsene Kanyamibwa, Manon Chédeville, Sherwin Nedaei Janbesaraei, Annette Horstmann

**Affiliations:** Department of Psychology, Faculty of Medicine, University of Helsinki, Helsinki, Finland

**Keywords:** food choice, hyperpalatable foods, diet, nutritional intelligence, Finland

## Abstract

Finnish adults do not simply gravitate toward hyperpalatable snacks: they prefer non-hyperpalatable options, which they perceive as healthier and more satiating. This finding challenges the dominant account of snacking as passive overconsumption driven primarily by hyperpalatability. It also addresses a key discussion in research: whether consumers’ everyday snack choices reflect implicit judgments about foods’ nutritional properties, rather than taste alone. Using the FinnFoodPics database, we found that participants accurately estimated snack energy density for hyperpalatable foods and showed consistent relationships among liking, expected satiation, and perceived healthiness for non-hyperpalatable foods. These results support a form of nutritional intelligence in everyday food choice. Furthermore, they suggest that food policies may be more effective when they consider snack consumption to be more complex than a simple model of hyperpalatable-food-driven intake.

Food powerfully reinforces behavior by generating reward. Decades of research show that modern energy-dense, hyperpalatable foods (HPFs) strongly drive eating behavior and disrupt homeostatic regulation [1]. Importantly, recent evidence suggests that the conscious sensory qualities of food, such as taste and smell, explain only part of excessive consumption [2]. Oral sensory cues create fleeting impressions of liking or disliking, serving as proxy rewards with short-lived effects on reinforcement [3]. The most potent influence emerges after ingestion, when the gut sends unconscious reward signals to the brain, sustaining overeating [2], [3].

This evidence portrays humans as largely reactive consumers shaped by a food environment that promotes overconsumption. At the same time, people do resist these drives and make deliberate food choices. This raises a central question: what happens when humans make deliberate judgments about food? Given that HPFs possess high reward value [1], [4], [5] and that both estimated and actual energy density (AED) influence food valuation and intake [6], [7], these factors exert considerable influence on meal size [8]. Although anticipatory evaluations, such as liking, cannot speak directly to reinforcement, they are nonetheless mechanistically linked to intake before consumption [6], [7], [8]. Building on this evidence, we examined whether people’s evaluations of familiar snack foods that differ in hedonic reward and energy density provide indirect, partial evidence of passive overconsumption.

In this study, we evaluated participants’ responses comparing HPF images (HPFs, *N* = 28) with non-HPFs (*N* = 44) using our tailored image database, FinnFoodPics [9]. We defined hyperpalatability quantitatively using the Fazzino et al. [5] criteria, which classify foods based on thresholds for the percentage of calories from fat, carbohydrates, and simple sugars, and for sodium weight (grams). Each food was rated on explicit liking, estimated energy density (EED), expected satiation, familiarity, frequency of consumption, and perceived healthiness. Hyperpalatability showed little effect on EED, with regression indicating a nonsignificant association (*β* = 0.43, *P* = 0.47; Table 1). Likewise, hyperpalatability groups did not differ in AED [*t*(1, 70) = –0.03, *P* = 0.97, BF_10_ = 0.24% ± 0.01%] or EED [*t*(1, 64) = 1.64, *P* = 0.1, BF_10_ = 0.71% ± 0.01%], suggesting that participants perceived no meaningful differences in EED between HPFs and non-HPFs. Notably, within-category correlation analysis revealed that participants estimated the energy density of HPFs accurately (*r* = 0.66, *P* = 1.5e-05, BF_10_ = 371.2% ± 0%; Figure 1A). In contrast, their estimates for non-HPFs were much less accurate (*r* = 0.094, *P* = 0.55, BF_10_ = 0.76% ± 0%; Figure 1B).

**TABLE 1:** Linear mixed models of EED, expected satiation, frequency of consumption, liking, and perceived healthiness with HPFs as the reference level of the predictor

Predictor (reference: HPF carbohydrates) (4464 observations for 72 food items ×; *N* = 62 participants = 4464 observations)	Unstandardized *β*	SE	*P* value	95% CI
Estimated energy density				
HPFs (intercept)	55.04	1.46	<0.001	52.1, 58
Non-HPFs	0.43	0.61	0.47	–0.76, 1.64
Combo (C+F)	9.23	0.71	<0.001	7.83, 10.6
Fat	9.24	0.71	<0.001	7.85, 10.6
Expected satiation				
HPFs (intercept)	35.39	1.82	<0.001	31.8, 38.9
Non-HPFs	3.63	0.67	<0.001	2.33, 4.94
Combo (C+F)	0.81	0.78	0.29	–0.71, 2.34
Fat	7.99	0.77	<0.001	6.47, 9.50
Frequency of consumption				
HPFs (intercept)	1.60	0.28	<0.001	1.05, 2.15
Non-HPFs	1.25	0.17	<0.001	0.91, 1.59
Combo (C+F)	0.29	0.20	0.15	–0.11, 0.69
Fat	2.04	0.20	<0.001	1.64, 2.43
Liking				
HPFs (intercept)	54.13	0.83	<0.001	52.5, 55.7
Non-HPFs	3.02	0.5	<0.001	2.05, 3.99
Combo (C+F)	6.55	0.58	<0.001	5.42, 7.69
Fat	3.45	0.58	<0.001	2.32, 4.58
Perceived healthiness				
HPFs (intercept)	36.34	1.41	<0.001	33.5, 39
Non-HPFs	6.22	0.82	<0.001	4.61, 7.83
Combo (C+F)	–12.40	0.96	<0.001	–14.2, –10.5
Fat	6.46	0.96	<0.001	4.59, 8.33

C+F, carbohydrate-fat combinations; EED, estimated energy density; HPF, hyperpalatable food.

**FIGURE 1:**
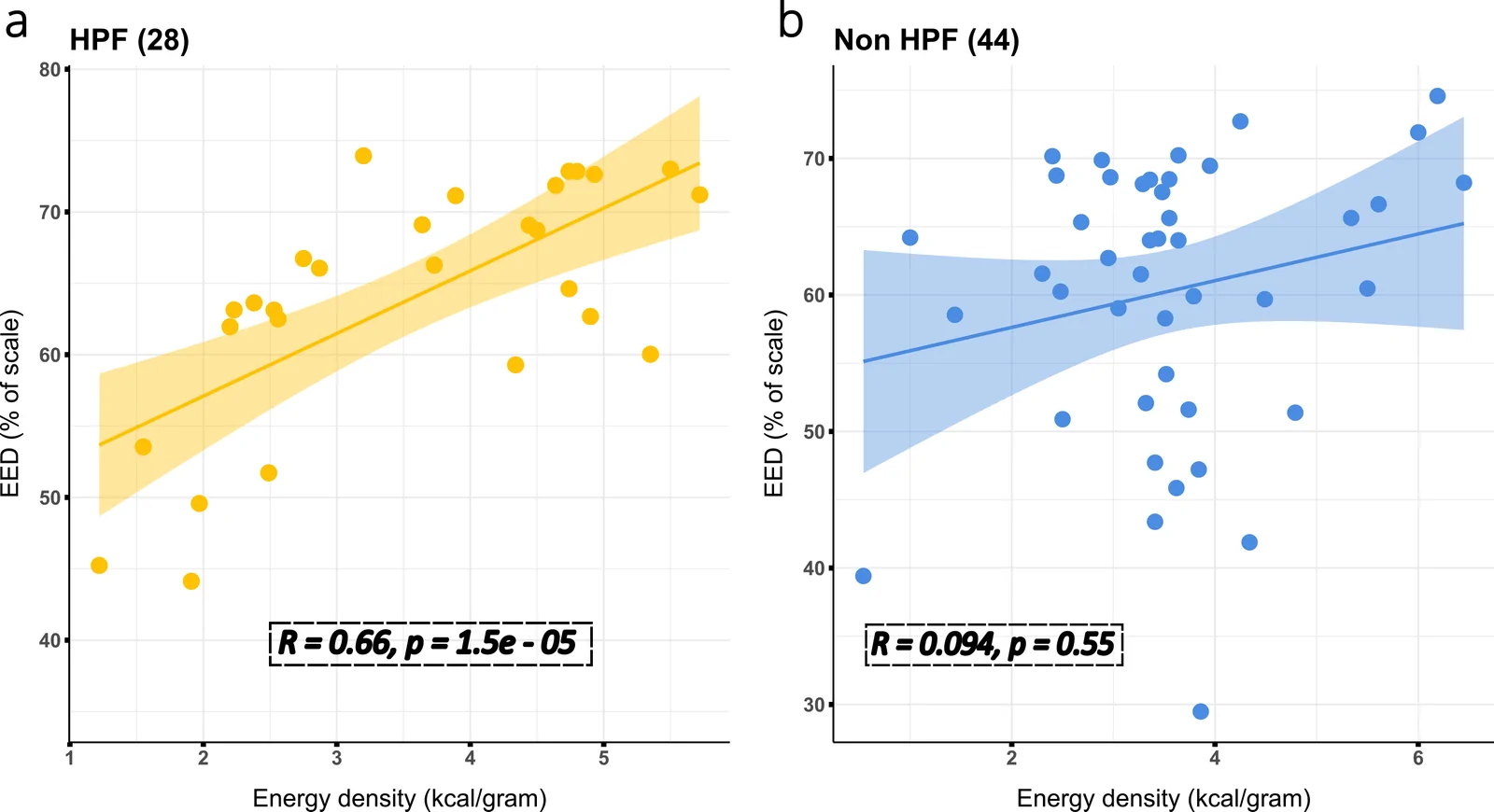
Energy density of foods (kcal/g) plotted against energy density as estimated by participants (EED, % of scale) for HPF (left) and non-HPF (right) items; lines show Spearman regression fits and 95% confidence intervals. EED, estimated energy density; HPF, hyperpalatable food.

We then investigated whether participants’ expected satiation aligned with their energy density ratings, testing if perceived fullness corresponds with energy estimates. Next, we examined whether consumption frequency reflected EED, AED, or satiation ratings, looking for signs that these perceptions influence dietary choices. In addition, we examined the potential influence of carbohydrate-fat combinations (C+F) compared with foods with most calories from fat or carbohydrates only.

We found that participants perceived non-HPFs as 3 times more satiating than HPFs (*β* = 3.63, *P* < 0.001) (Table 1). Consumption frequency also differed: participants reported 1.24 additional consumption days per month for non-HPFs compared with HPFs (*β* = 1.24, *P* < 0.001) (Table 1). Although this trend suggests that expected satiation may influence how frequently participants choose these foods, it is well established that AED shapes both satiation and consumption patterns [10]. Furthermore, the proportion of energy derived from HPFs and protein content are known to affect ad libitum intake [8]. Given that AED was comparable between HPF and non-HPF choices in our sample (Supplemental Table 1), despite non-HPFs containing more protein (1.62 times; *P* = 0.02), these findings highlight the need for caution in drawing causal inferences about the relationship between satiation, AED, and consumption frequency in this context.

Additionally, we explored whether HPFs, characterized by their sugar, fat, and sodium content [5], are tied to liking, usually associated with pleasant taste [11], and potentially promote intake. Contrary to expectation, participants rated non-HPFs as more palatable than HPFs, with mean liking scores 3 points higher (on a 0%–100% of a labeled hedonic scale) (*β* = 3.01, *P* < 0.001) (Table 1). This outcome corresponds to a small (*d* = –0.04, Supplemental Table 1) but reliable difference in hedonic rating. In addition, it challenges the assumption that HPFs are universally preferred and instead indicates a tendency to report greater liking for non-HPFs in familiar snacking contexts. Moreover, consistent with their higher liking ratings, participants reported eating HPFs 2 to 3 days per month (mean) compared with 3 to 4 d for non-HPFs (Supplemental Table 1). Importantly, familiarity ratings did not differ between HPFs and non-HPFs [*t*(51.19) = −1.28, *P* = 0.2; Supplemental Table 1], suggesting that familiarity is unlikely to drive these findings.

Nutritive values (e.g., fat, sugar), price [12], portion size [13], fiber [14], and protein content [15] all influence human food choices. Thus, we hypothesized that these attributes might differ between food groups, along with perceived healthiness. All the images contained 120 kcal of food with varying portion sizes (g/120 kcal) and actual retail prices compiled from local supermarkets [9]. Participants rated non-HPFs as 6 times healthier than HPFs (*β* = 6.21, *P* < 0.001) (Table 1). No significant difference was observed for portion and price, but non-HPFs contained more fiber (0.68 times; *P* = 0.01) with medium effect sizes (Supplemental Table 1). HPFs contained more fat and sodium than non-HPFs (both *P* ≤ 0.02), with similar carbohydrate and sugar contents (*P* ≥ 0.09), so differences in liking, satiation, and perceived healthiness may reflect these compositional differences (i.e., hyperpalatability classification; Supplemental Table 1).

In this sample, perceptual evaluations did not show systematic underestimation of nutritive qualities or preferential valuation of HPFs relative to non-HPFs, suggesting that, at least for familiar snacks, participants’ ratings are compatible with the notion of nutritional intelligence [16] although alternative interpretations remain possible. The high ratings for perceived healthiness among non-HPFs may reflect learned associations acquired through repeated exposure and cultural knowledge, although the present study did not assess this directly. Links between frequent HPF exposure, blunted pleasure responses, and risk of diet-related diseases have been reported [1], [5]. Moreover, given that humans are not consistently reliable at detecting taste differences [17], the higher expected satiation and reported consumption frequency for non-HPFs suggest that factors beyond immediate reward contribute to food evaluation without implying a specific causal mechanism. Interestingly, despite the expectation that HPFs may be more liked because of their sugar, fat, and sodium content, participants favored non-HPFs, echoing studies that blunted liking may develop through repeated exposure to HPFs [1], [3], [12]. Over time, the diminished sensation of fullness may reduce liking, matching, or even favoring non-HPFs for some individuals. In this context, these findings are compatible with prior evidence suggesting that oral sensory properties alone are insufficient to explain excessive food intake [2]. However, they remain surprising as Finland ranks very high in candy (5.8 kg/capita) and chocolate consumption (7.1 kg/capita), added sugar (>10% of energy intake), and sodium intake (>1.5–2 g/d) [18], [19].

Further, the carbohydrate-to-fat ratio in energy to satiety remains a strong determinant of food liking [2], [20]. Our analysis showed that more HPFs (18 items) combined carbohydrates and fat compared with non-HPFs (11 items), and items with this combination were generally rated as more liked (*β* = 6.55, *P* < 0.001), yet this did not translate into a stronger liking for HPFs overall [*t*(52.2) = –0.20, *P* = 0.84; Supplemental Table 1].

Our findings are based on a small sample (*N* = 62) of young, healthy, highly educated adults (M = 15 years of education) [9]. Accordingly, the results should be interpreted as preliminary and context-specific, as they may not generalize to individuals with lower educational attainment. The study also relied on snack images rather than real meals, which could influence ratings; future studies should incorporate actual intake measurements and broader samples. Snack intake is nonetheless directly related to BMI and weight gain [21]. Finally, the study did not measure the wanting aspect of food reward, potentially overlooking the complex interplay between wanting and liking that more accurately reflects postingestive signals and food valuation.

To conclude, although our data come from a narrow demographic, they are consistent with the view that nutritional intelligence in humans may be underestimated in laboratory settings [16]. As previous work shows, humans do not make mindless food decisions throughout the day [22], our results are consistent with the possibility that individuals can make informed evaluations of familiar foods. Even though, in Finland, researchers have highlighted challenges in interpreting nutrition recommendations at the population level [23], this does not preclude context-dependent sensitivity to familiar food attributes at the individual level.

Our exploratory analyses suggest that within this sample, food valuation in familiar environments reflects an interaction between cultural knowledge and biological reward processes, albeit only the hedonic component, rather than reward in the motivational sense [16]. More research using designs that directly link perceptual evaluation to real-life choice and intake is required. However, considering the importance of eating behavior for healthy aging [24], future studies should consider this complexity when designing experiments and interpreting findings, without assuming hyperpalatability uniformly overrides learned food evaluation. Additionally, differences between countries in food availability, cultural norms, food marketing, and familiarity may influence evaluations of HPFs. Thus, broader cross-cultural research should be considered alongside individual-level approaches to account for local cuisine and generalize across countries. Rather than focusing solely on individual responsibility, it is important to recognize that humans rely on learned cues and environments to guide food choices. Thus, we should refrain from requiring citizens to become nutrition experts—humans never were.

## Methods

This was a secondary analysis of data collected to establish FinnFoodPics [9]. Participants rated FinnFoodPics on a continuous scale across 7 variables, for example, liking [9]. The image database was organized into 3 food-item categories, with most calories coming from 1 or 2 macronutrients (carbohydrate, fat, and carbohydrate + fat or combo) [9]. We used a published quantitative definition to define the HPF and non-HPF groups [5] (Supplemental Figure 1). We recruited 62 healthy participants (36 female, 26 male; mean age 25.96 ± 6.70 y; mean BMI 23.78 ± 3.54 kg/m^2^) from the University of Helsinki and the greater Helsinki area. Exclusion criteria included dietary restrictions for any reason (religious, personal choice, or health-related) as well as active dieting or intentional caloric restriction at the time of testing. Additionally, participants reported a mean Restrained Eating score of 31 ± 4.23 (range 24–40), suggesting a moderate baseline tendency toward conscious dietary control [9]. Habitual dietary fat and sugar intake was moderate (total score 55.35 ± 6.29, range 39–74), indicating that participants’ everyday consumption patterns were not skewed toward unusually low or high habitual intake [9]. Emotional eating scores (29.35 ± 9.22, range 13–63) reflected typical variability, suggesting that emotionally driven consumption patterns were not systematically overrepresented or underrepresented in the sample [9]. External eating scores (21 ± 7.33, range 10–35) indicated moderate sensitivity to food-related environmental cues (e.g., sight of food) [9]. We recorded all food ratings on continuous vertical hedonic (liking) and horizontal scales (Supplemental Table 2). Descriptive statistics and t tests for the image visual properties are shown in Supplemental Table 3. Labels for images are shown in Supplemental Table 2.

We performed all statistical analyses using R (RStudio version 4.5.1) and JASP Team (JASP version 0.17.3.0). We conducted linear mixed-effects models (LMMs), t tests, and correlations to investigate EED, AED, liking, frequency of consumption, expected satiation, and healthiness in relation to hyperpalatability. The LMMs included fixed effects for hyperpalatability (HPF compared with non-HPF) and macronutrient category (carbohydrate, fat, carbohydrate + fat), allowing examination of how these factors influence our variables across conditions. We specified a random intercept for each participant to account for individual variability.

## Ethical statements

This study was conducted in compliance with the principles of the Declaration of Helsinki and was reviewed and deemed ethically acceptable by the Ethical Review Board in Humanities and Social and Behavioural Sciences of the Medical Faculty at the University of Helsinki (Statement 35/2022). All participants provided written informed consent before participation and were compensated for their time.

## Author contributions

The authors’ responsibilities were as follows – AK: contributed to conceptualization, methodology, writing original draft, formal analysis, investigation, data curation, software, visualization, resources, writing—review and editing, funding acquisition, and project administration; MC: contributed to data curation, validation, software, and writing—review and editing; SNJ: contributed to methodology, software, validation, and writing — review and editing; AH: contributed to conceptualization, methodology, supervision, project administration, and writing—review and editing; and all authors: read and approved the final manuscript.

## Data availability

The final 72-item list is publicly available, and the code produced for this manuscript is openly available at Github:https://github.com/ArseneDK/Nutrititional_Intelligence_Finland.

## Declaration of Generative AI and AI-assisted Technologies in the Writing Process

During the preparation of this work, AK used Claude AI to improve readability and language. Afterward, AK reviewed and edited the content as needed and takes full responsibility for the publication’s content.

## Funding

AK was supported by funding from the Jenny and Antti Wihuri Foundation. AH and SNJ were supported by the Stiftelsen Kristian Gerhard Jebsen within the K.G. Jebsen Centre for Obesity Research (CORA). MC was funded by the University of Helsinki, as part of the ICANDOC Doctoral Education Pilot Precision Cancer Medicine.

## Conflict of interest

The authors report no conflicts of interest.
