# Supplementary material for: Nutritional Intelligence? Hyperpalatable Foods Do Not Dominate Evaluations of Familiar Snacks in a Finnish Sample
Source: Curr Dev Nutr. 2026 Oct 9;10(11):109553. doi: 10.1016/j.cdnut.2026.109553 (PMC13738118; doi:10.1016/j.cdnut.2026.109553)
Supplement: Supplementary file 1 [file mmc1.docx]

Supplementary material

# Nutritional intelligence? Hyper-palatable foods do not dominate evaluations of familiar snacks in a Finnish sample

Arsene Kanyamibwa^1^, Manon Chédeville^1^, Sherwin Nedaei Janbesarei^1^, Annette Horstmann^1^

1. Department of Psychology, Faculty of Medicine, University of Helsinki, Helsinki, Finland

# Table S1: Descriptive statistics and independent-samples Welch t-test for portion size, actual price, protein, and fibre content between HPFs and non-HPFs

|  | **HPF (28)** | **Non-HPF (44)** | **Group** | |
| --- | --- | --- | --- | --- |
|  | **Mean ± SEM, Range** | **Mean ± SEM, Range** | **Welch t-test** | **Cohen’s d** |
| **Energy density (kcal/g)** | 3.56 ± 0.25, 1.22 - 5.72 | 3.57 ± 0.18, 0.54 - 6.45 | t(54.5) = -0.032 , p = 0.97 | d = -0.008 |
| **Portion (g/120 kcal)** | 39.5± 3.55, 20.9 - 98.3 | 41.5± 4.94, 18.6 - 222.2 | t(69.4) = -0.33 , p = 0.73 | d = -0.07 |
| **Protein (g/120 kcal)** | 2.66± 0.35, 0.46 - 7.09 | 4.29± 0.59, 0 - 21.7 | t(66) = -2.34, p = **0.02** | d = -0.52 |
| **Fibre (g/120 kcal)** | 0.37± 0.13, 0 - 2.95 | 1.05± 0.24, 0 - 6.14 | t(64.4) = -2.43, p = **0.01** | d = -0.54 |
| **Carbohydrate (g/120 kcal)** | 11.62± 1.46, 0 - 23.6 | 15.29± 1.56, 0- 28.8 | t(68.2) = −1.71, p = 0.09 | d = -0.40 |
| **Fat (g/120 kcal)** | 6.66± 0.6, 0.62 - 13.1 | 4.32± 0.55, 0 - 28.8 | t(63.4) = 2.83, p = **0.006** | d = 0.67 |
| **Sugars (g/120 kcal)** | 6.08± 1.07, 0 - 18.9 | 5.89± 1.33, 0 - 28.8 | t(70.0) = 0.11, p = 0.91 | d = 0.02 |
| **Sodium (g/120 kcal)** | 0.21± 0.04, 0 - 0.96 | 0.09± 0.01, 0 - 0.23 | t(30.9) = 2.52, p = **0.017** | d = 0.66 |
| **Price (Euros/120 kcal)** | 0.36± 0.02, 0.13 - 0.81 | 0.46± 0.07, 0.03 - 2.5 | t(55.5) = -1.36, p = 0.17 | d = -0.29 |
| **Liking (% of scale)** | 59.4± 1.11, 47.9 - 70.1 | 59.7± 0.78, 48.9 - 72.1 | t(52.2) = -0.20, p = 0.84 | d = -0.04 |
| **Familiarity (% of scale)** | 70.7± 1.84, 48.7 - 87.4 | 73.6± 1.26, 48.7 - 85.8 | t(51.19) = −1.28, p = 0.20 | d = -0.31 |
| **Frequency of consumption (days/month)** | 2.44± 0.36, 0.22 - 7.11 | 3.48± 0.4, 0.22 - 10.8 | t(68.84) = −1.89, p = 0.06 | d = -0.43 |

# Table S2: Questions and labels for the food ratings.

| **Subjective Variable** | **Question** | **Labels** |
| --- | --- | --- |
| **Liking** | How much do you like this food? | Most disliked sensation imaginable,  Most liked sensation imaginable (end-to-end labels) |
| **Familiarity** | How familiar is this food? | Extremely unfamiliar, Extremely familiar |
| **Frequency of consumption** | How often do you eat this food? | <1x per month, 2-3x per month, 1-2x month, 3-4x per month, 5+x per month |
| **Perceived healthiness** | How healthy is this food? | Extremely unhealthy, Extremely healthy |
| **Expected satiation** | How filling do you expect this food portion to be? | Not filling at all,  Extremely filling |
| **Estimated energy content** | How many calories are in this portion? | 0, 60, 120, 180, 240 kcal |
| **Estimated energy density** | How energy dense is this food? | Extremely low,  Extremely high |

# Table S3: Descriptive statistics and Independent-samples Welch t-tests for visual properties

|  | **HPF (28)** | **Non-HPF (44)** | **Group** | |
| --- | --- | --- | --- | --- |
|  | **Mean ± SEM, Range** | | **Welch t-test** | **Cohen’s d** |
| **Visual area of food (cm^2^)** | **49.4**± 4.98, 20-120 | 83.9± 9.78, 5.54-315 | t(61.6) = -3.14, p = **0.003** | d = -0.69 |
| **Red** | 0.34± 0.00, 0.33-0.34 | 0.34± 0.00, 0.33-0.35 | t(67.4) = -2.87, p = **0.005** | d = -0.64 |
| **Green** | 0.33± 0.00, 0.335-0.339 | 0.33± 0.00, 0.33-0.34 | t(68.9) = -1.23, p = 0.22 | d = -0.28 |
| **Blue** | 0.32± 0.00, 0.31-0.32 | 0.32± 0.00, 0.30-0.32 | t(65.3) = 2.83, p = **0.006** | d = 0.63 |
| **Brightness** | 59.3± 0.6, 54.3-66.2 | 60.4± 0.81, 46.4-72.1 | t(69.7) = -1.06, p = 0.28 | d = -0.24 |
| **Within-Object Contrast** | 29.4± 0.8, 22.8-43.2 | 28.8± 0.77, 21.6-45.1 | t(65.2) = 0.53, p = 0.59 | d = 0.12 |
| **Spatial frequency** | 16.6± 0.06, 15.8-17.1 | 16.7± 0.09, 15.5-18.3 | t(68.5) = -0.59, p = 0.55 | d = -0.13 |
| **Complexity** | 0.02± 0.00, 0.01-0.03 | 0.02± 0.001, 0.01-0.06 | t(69.5) = -2.43, p = 0.01 | d = -0.55 |

# Figure S1: Example images for HPF and non-HPF food items

**Figure S1**. Selection of *FinnFoodPics* images with corresponding food names. Items were grouped into hyperpalatable and non-hyperpalatable food items. All images were color photographs measuring 4252 × 2829 pixels at a resolution of 300 dots per inch (dpi).
